# The Molecular Nature of Redox-Conductive Metal–Organic Frameworks

**DOI:** 10.1021/acs.accounts.4c00430

**Published:** 2024-09-17

**Authors:** Jingguo Li, Sascha Ott

**Affiliations:** Wallenberg Initiative Materials Science for Sustainability, Department of Chemistry, Ångström Laboratory, Uppsala University, Box 523, 75120 Uppsala, Sweden

## Abstract

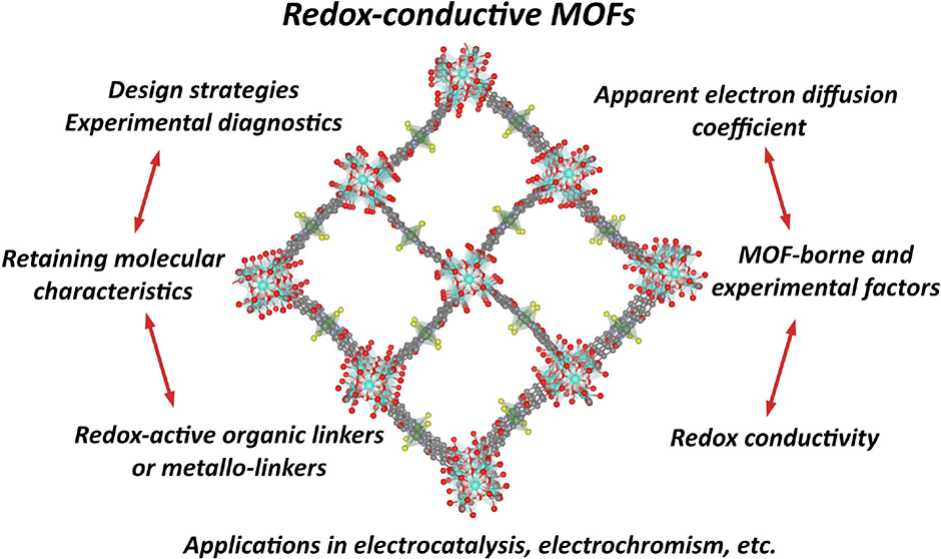

Redox-conductive metal–organic frameworks (RC-MOFs) are a class of porous materials that exhibit electrical conductivity through a chain of self-exchange reactions between molecularly defined, neighboring redox-active units of differing oxidation states. To maintain electroneutrality, this electron hopping transport is coupled to the translocation of charge balancing counterions. Owing to the molecular nature of the redox active components, RC-MOFs have received increasing attention for potential applications in energy storage, electrocatalysis, reconfigurable electronics, etc. While our understanding of fundamental aspects that govern electron hopping transport in RC-MOFs has improved during the past decade, certain fundamental aspects such as questions that arise from the coupling between electron hopping and diffusion migration of charge balancing counterions are still not fully understood.

In this Account, we summarize and discuss our group’s efforts to answer some of these fundamental questions while also demonstrating the applicability of RC-MOFs in energy-related applications. First, we introduce general design strategies for RC-MOFs, fundamentals that govern their charge transport properties, and experimental diagnostics that allow for their identification. Selected examples with redox-active organic linkers or metallo-linkers are discussed to demonstrate how the molecular characteristics of the redox-active units inside RC-MOFs are retained. Second, we summarize experimental techniques that can be used to characterize charge transport properties in a RC-MOF. The apparent electron diffusion coefficient, *D*_*e*_^*app*^, that is frequently determined in the field and obtained in large perturbation, transient experiments will be discussed and related to redox conductivity, σ, that is obtained in a steady state setup. It will be shown that both MOF-intrinsic (topology, pore size, and apertures) and experimental (nature of electrolyte, solvent) factors can have noticeable impact on electrical conductivity through RC-MOFs. Lastly, we summarize our progress in utilizing RC-MOFs as electrochromic materials, materials for harvesting minority carriers from illuminated semiconductors and within electrocatalysis. In the latter case, recent work on multivariate RC-MOFs in which redox active linkers are used to “wire” redox catalysts in the crystal interiors will be presented, offering opportunities to independently optimize charge transport and catalytic function.

The ambition of this Account is to inspire the design of new RC-MOF systems, to aid their identification, to provide mechanistic insights into the governing ion-coupled electron hopping transport mode of conductivity, and ultimately to promote their applications in existing and emerging areas. With basically unlimited possibilities of molecular engineering tools, together with research in both fundamental and applied fields, we believe that RC-MOFs will attract even more attention in the future to unlock their full potential.

## Key References

- JohnsonB. A.; BhuniaA.; FeiH.; CohenS. M.; OttS.Development of a UiO-Type Thin Film Electrocatalysis Platform with Redox-Active Linkers. J. Am. Chem. Soc.2018, 140 ( (8), ), 2985–2994.29421875 10.1021/jacs.7b13077PMC6067658(1) This work represents a significant advancement in the development of organic linker based redox-active MOFs and MOF thin films.
- RoyS.; HuangZ.; BhuniaA.; CastnerA.; GuptaA. K.; ZouX.; OttS.Electrocatalytic Hydrogen Evolution from a Cobaloxime-Based Metal-Organic Framework Thin Film. J. Am. Chem. Soc.2019, 141 ( (40), ), 15942–1595031508946 10.1021/jacs.9b07084PMC6803166.^2^ This study reports the design of metallo-linker based redox-conductive MOFs and explores their electrocatalytic activity toward hydrogen evolution reaction.
- CastnerA. T.; SuH.; Svensson GrapeE.; IngeA. K.; JohnsonB. A.; AhlquistM. S. G.; OttS.Microscopic Insights into Cation-Coupled Electron Hopping Transport in a Metal–Organic Framework. J. Am. Chem. Soc.2022, 144 ( (13), ), 5910–5920.35325542 10.1021/jacs.1c13377PMC8990995(3) This work systematically investigates the effect of counterion size, solvent polarity, and ion pairing on electron hopping transport in redox-conductive MOFs.
- LiJ.; KumarA.; JohnsonB. A.; OttS.Experimental manifestation of redox-conductivity in metal–organic frameworks and its implication for semiconductor/insulator switching. Nat. Commun.2023, 14 ( (1), ), 4388.37474545 10.1038/s41467-023-40110-6PMC10359279(4) This study presents direct experimental evidence of the bell-shaped redox conductivity in redox-conductive MOFs.
- LiJ.; KumarA.; OttS.Diffusional Electron Transport Coupled to Thermodynamically Driven Electron Transfers in Redox-Conductive Multivariate Metal–Organic Frameworks. J. Am. Chem. Soc.2024, 146 ( (17), ), 12000–12010.38639553 10.1021/jacs.4c01401PMC11066865(5) This study demonstrates the coexistence of diffusional electron hopping transport between linkers of the same type and mediated electron transfer between linkers of different types in multivariate redox-conductive MOFs.

## Introduction

Conductive metal–organic frameworks (MOFs) that permit electron transport through their porous structures continue to receive considerable interest due to their distinct spectrum of applications including energy storage, electrocatalysis, reconfigurable electronics, etc.^6−11^ Unlike conventional MOFs that are composed of hard-metal-based secondary building units (SBUs) and redox-inactive polydentate organic linkers, conductive MOFs are generally built by SBUs or organic linkers that are electroactive. The mode of charge transport in a conductive MOF depends fundamentally on whether it retains the “molecular” properties of individual building blocks or exhibits “collective” properties of a solid-state material instead. Correspondingly, two different conduction mechanisms, electron hopping transport and band-type transport, have to be considered,^10^ and correct understanding of the charge transport mechanism in conductive MOFs is key for their future deployment. The band-type transport in MOFs is rooted in the traditional band structure models of solid-state materials with significant band dispersion. In the absence of any band dispersion, electrons can be transported through electronically isolated redox active units by a series of hopping events.

This Account focuses on electroactive MOFs that operate through the redox-hopping mechanism, and we will refer to such systems as redox-conductive MOFs (RC-MOFs). As mentioned above, the fundamental requirement for RC-MOFs is that the redox-active units should be electronically isolated. Thin films of RC-MOFs that are directly grown on conducting substrates are most suitable to investigate hopping transport phenomena as charge transport can be treated as a one-dimensional diffusion problem normal to the substrate surface.^1−5,12,13^ Alternative electrode architectures in which bulk MOF particles are immobilized on conducting substrates, for example, by drop-casting of MOF inks that contain nafion-type binders and carbon additives to increase conductivity, often exhibit large capacitive currents and generally impede the extraction of quantitative data.

**Figure 1:**
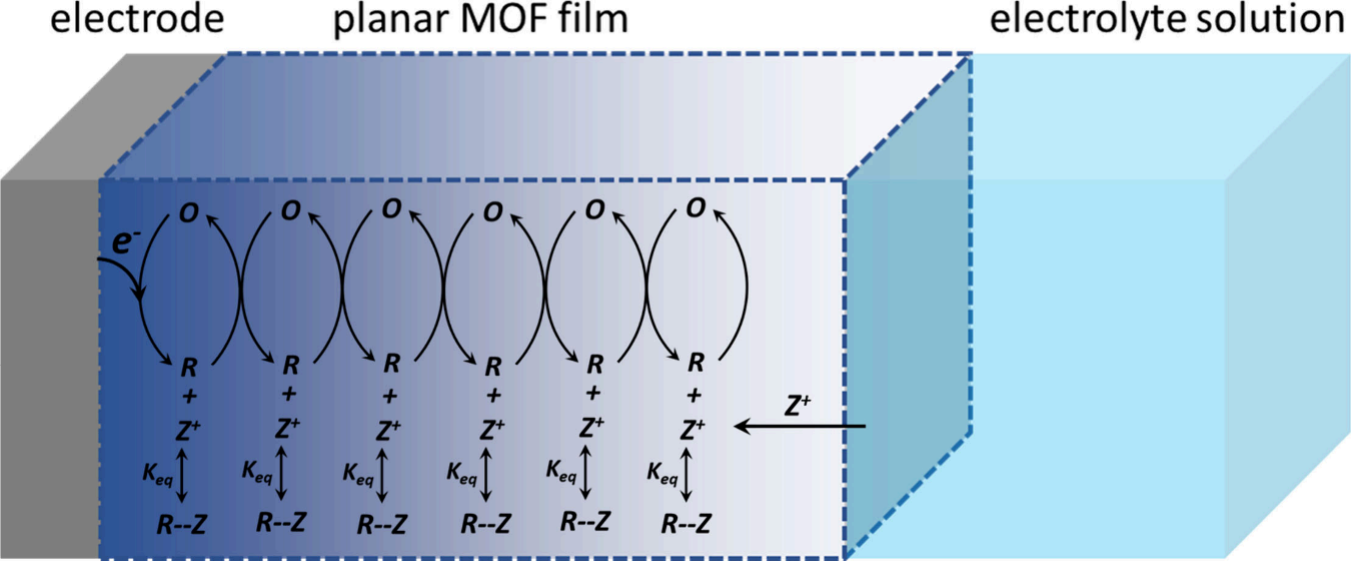
Schematic representation of diffusional electron hopping transport in an RC-MOF film grown on an electrode surface after a large step potential experiment. Electron injection at the electrode-MOF interface transforms electronically isolated redox-active species **O** to **R**, and meanwhile, charge balancing counterions **Z**^**+**^ are moving from the electrolyte solution into the MOF film to maintain overall charge neutrality. The color gradient inside the MOF film represents the concentration gradient of **R**.

The general principles of electron hopping transport in RC-MOFs with redox-active linkers are exemplified on a cathodic process in Figure 1. Applying an electrochemical potential that is sufficiently negative to drive electron transfer from the substrate to the redox active unit, as done in chronoamperometry or fast scan cyclic voltammetry (CV) experiments, results in a high population of reduced linkers close to the MOF/substrate interface. A concentration gradient of reduced linkers will evolve in the bulk of the film, and it is this concentration gradient that drives the transport of electrons. The situation is identical to that in a homogeneous electrochemistry experiment where reduced analytes are created at the electrode-electrolyte interface and diffuse into the bulk of the solution. The difference in RC-MOFs is that the reduced linker is not physically diffusing, but the electron diffuses by discrete hopping events between immobilized redox-active sites. In addition, electron diffusion in an RC-MOF has a physical boundary that is given by the finite thickness in the MOF film (*d*_*f*_). If the electrochemistry experiment is conducted on fast time scales (as, for example, defined by the scan rate,ν, in a CV experiment), only the bulk of the RC-MOF film is probed, and electron diffusion is in the semi-infinite regime. Contrary, at slow scan rates, the finite limits of the film are explored. Importantly, the addition of electrons (or removal for oxidative processes) from the redox-active units is always coupled to the translocation of a charge compensating counterions from the electrolyte (Figure 1).

In this Account, we first discuss the design principles of RC-MOFs with a few examples. Then, we will introduce ways to evaluate charge transport properties in RC-MOFs, either through transient state experiments for the apparent electron diffusion coefficient, *D*_*e*_^*app*^, or through steady state experiments for the redox conductivity, σ. We will discuss the similarities and differences between these two metrics and assumptions (and limitations) that go into these models. Intrinsic and extrinsic factors that will influence the charge transport rate will be discussed with selected examples, and potential applications of RC-MOFs will briefly be discussed.

## RC-MOFs: Characteristics, Diagnostics, and Design

**Figure 2:**
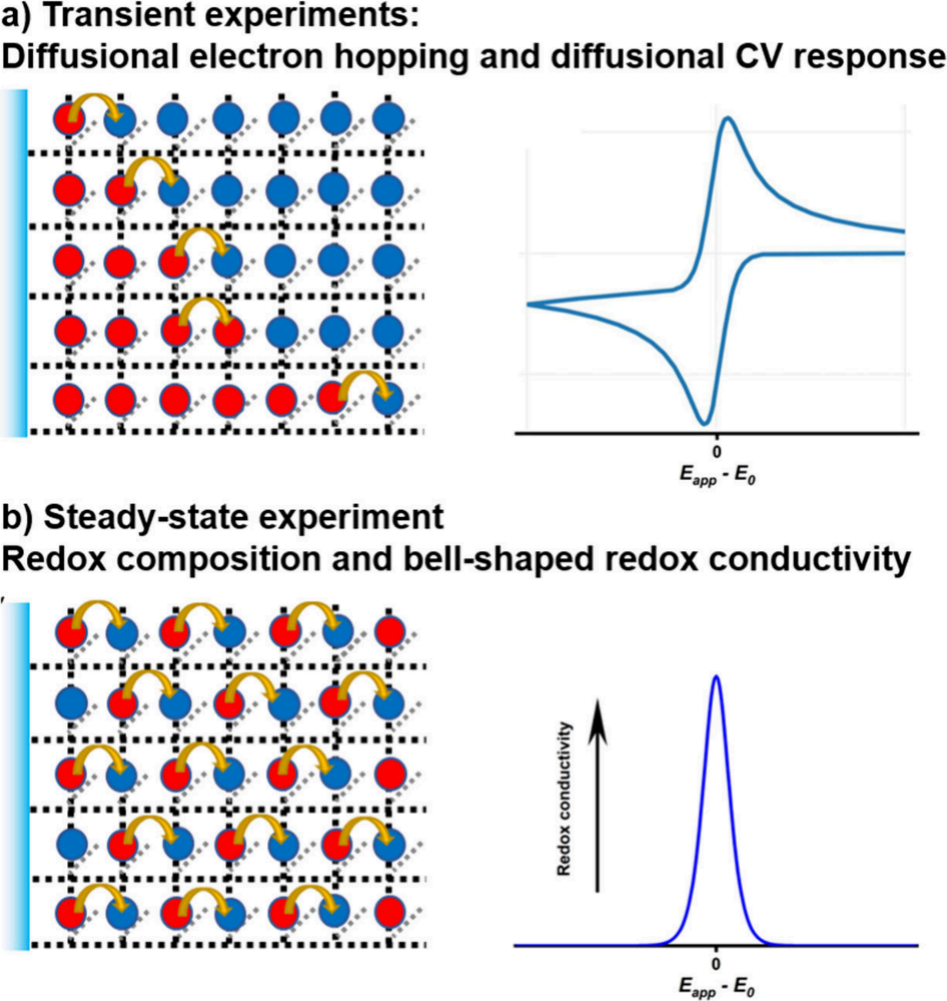
Schematic representation of diffusional electron hopping transport within a RC-MOF in transient electrochemical experiments, and a “molecular” CV profile (a). Schematic representation of an RC-MOF with a 50:50 distribution of reduced and oxidized linkers obtained by applying a potential that corresponds to the *E*_*0*_ of the redox active component for an extended period of time. The 50:50 steady state redox composition of the film at its *E*_*0*_ is a direct outcome of the Nernst equation. Deviating from the *E*_*0*_ will alter the redox composition of the film and lead to a drop in redox conductivity. Consequently, a bell-shaped redox conductivity curve as a function of applied potential is observed for a RC-MOF (b).

To design RC-MOFs, electron delocalization between linkers and secondary building units (SBUs), and electronic inter-linker and inter-SBU interactions in all relevant oxidation states have to be minimized. In other words, through-bond, extended conjugation and through space charge delocalization that contributes to the band conduction mechanism need to be avoided.^10^ Essentially, the redox-active units that facilitate electron hopping inside the RC-MOF have to behave like discrete molecular species. Practically, this can be enforced by the use of redox-inert metal cations such as Zn^2+^ or Zr^4+^ in the SBUs. Experimentally, there are some diagnostics that can easily be applied as indicators for whether a MOF is redox-conductive or not, preferably on thin film samples.^1,2,4,5,14^ First, the redox-active units that are installed in a RC-MOF should retain the distinct electronic transitions of the individual molecular species in homogeneous phase in relevant protonation states. Second, the CV of a RC-MOF should have “molecular” Faradaic features, such as the classic “duck-shaped” response in Figure 2a, at a potential similar to that of the redox-active species in homogeneous solution.

Another diagnostic signature of an RC-MOF is the bell-shaped redox conductivity curve as a function of the potential applied to the electrode (Figure 2b). Such a phenomenon has been previously observed in related redox-active polymers,^15,16^ but was only recently reported in a MOF.^5^ The diagnostic redox conductivity profile can be extracted from relatively easy-to-perform electrochemical impedance spectroscopy studies. The bell-shaped redox conductivity curve is a direct result of the molecular nature of the contributing redox active components, as the possibility of a successful electron hopping event in RC-MOFs is fundamentally determined by the presence of electrons (or holes) localized on the linkers and the availability of neighboring acceptor (or donor) sites. Maximum redox conductivity, σ, is expected when the electron donor and electron acceptor “hopping sites” are in a 50:50 ratio (Figure 2b),^17^ which is the case at the midpoint potential of the redox-active unit according to the Nernst equation. When the applied potential deviates from the standard potential, the ratio of either the oxidized or reduced sites will decay quickly, as defined by the redox equilibria, and the electron hopping channels will be closed as well. The bell-shaped distribution of redox conductivity clearly discriminates whether an electroactive MOF is following primarily a band conduction or redox-hopping conduction mechanism.

**Figure 3:**
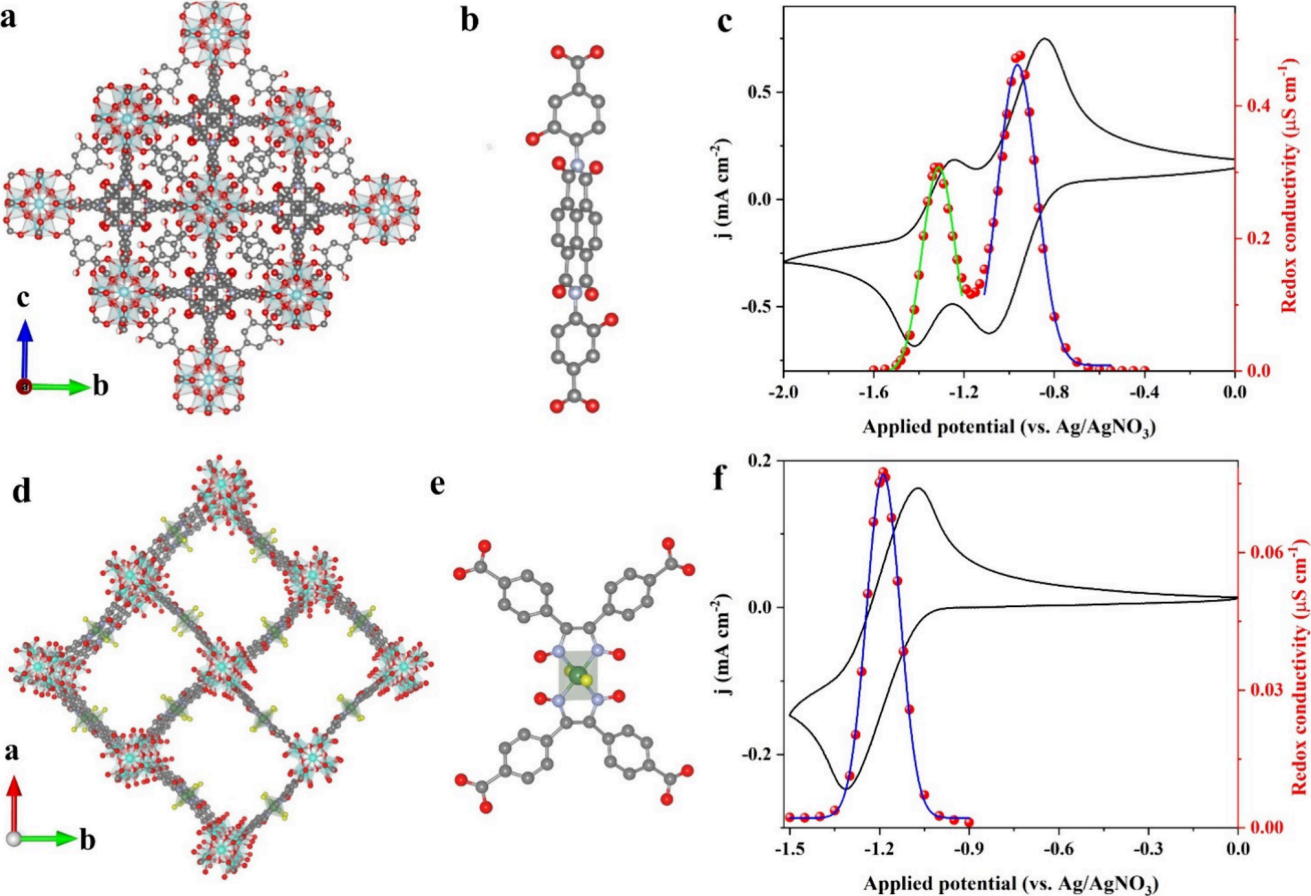
Crystal structure of Zr(NDI) (a) and UU-100(Co) (d) as determined by microcrystal electron diffraction and the molecular structure of corresponding NDI (b) and cobaloxime linker (e). CVs of thin films grown on FTO substrates (in black), and the corresponding redox conductivity curves, as deduced from electrochemical impedance spectroscopy of Zr(NDI) (c) and UU-100(Co) (f). The “molecular” CV waves, and the bell-shaped conductivity curves are characteristic of RC-MOFs. Reproduced from ref (4). Available under a CC-BY 4.0 license. Copyright 2023 Springer Nature.

While either the SBU or the linker may in theory be the redox active component in an RC-MOF, most studies are done on systems with redox-active organic linkers or metallo-linkers and redox-silent SBUs. So far, we are not aware of any established RC-MOFs that are built on metal-based SBUs according to the definition above, probably due to either the difficulty in retaining the discrete behavior of metal sites in SBUs^18,19^ or lack of detailed characterizations. In the case of RC-MOFs based on organic linkers, we reported a hexanuclear Zr^4+^ cluster SBU-based system with redox-active naphthalene diimide (NDI)-based linkers (Figure 3a,b).^1^ Such a MOF combines the advantages of the UiO-type structure,^20^ i.e. excellent chemical stability, high porosity and surface area, and the redox-active properties of the NDI linker. Zr(NDI) can be grown as thin films on FTO surfaces, which allows the interrogation of the MOF by UV/vis spectroscopy, electrochemistry, combinations thereof, and redox conductivity measurements. Using these techniques, it is shown that the NDI linkers behave in analogy to their homogeneous reference compounds, exhibiting, for example, two reversible one-electron redox processes that are arise from the expected NDI/NDI^•–^ and NDI^•–^/NDI^2–^ couples (Figure 3c). Moreover, the two bell-shaped redox conductivity curves that maximize at the midpoint potential of the two redox waves are observed. With these characteristics, it is beyond the shadow of a doubt that Zr(NDI) is a RC-MOF.

Redox conductivity in a MOF with a metallo-linker was demonstrated in 2019 when UU-100(Co) (UU = Uppsala University) that consists of tetradentate cobaloxime-tetra-phenylcarboxylate linkers (Figure 3d,e) and the same hexanuclear Zr^4+^ cluster SBU as in Zr(NDI) was reported.^2^ The crystal structure of UU-100(Co) was determined by transmission electron microscopy, revealing a tetragonal unit cell (*a* = *b* = 27.3 Å, and *c* = 19.6 Å) with a *P*4/*mbm* space group (Figure 3d). Such a structure effectively separates cobalt centers between neighboring linkers, similar to the case of the pyrene-based linkers in NU-901.^21^ When directly grown on FTO, both the electrochemical and electronic properties of UU-100(Co) can be easily assessed. It was shown that the half-wave potential of the reversible Co^II/I^ redox couple in the UU-100(Co) film is very similar to that of the free cobaloxime reference. UV–vis spectroelectrochemistry also confirmed the molecular electronic transitions of cobaloximes in the UU-100(Co) film, as indicated by characteristic absorptions of Co^I^ species at ∼520 and 670 nm. Finally, a characteristic bell-shaped redox conductivity profile was established at an applied potential of the Co^II/I^ redox couple (Figure 3f),^4^ making UU-100(Co) another clear-cut example of a RC-MOF.

## Diffusional Hopping Transport in RC-MOFs

### Transient Charge Transport Models and Their Limitations

As introduced above, charge transport in an RC-MOF operates through a series of ion-coupled electron hopping processes between neighboring linker sites. The nature of this process can be understood by various physical models of differing degrees of complexity.^16^ At the macroscopic level, it is generally assumed that the applied potential drops within the electrical double layer at the electrode-MOF interface, and there is enough counterion infiltration so that no electric field is present in the bulk MOF film. Further, when the existence of intermolecular interactions between redox-active species themselves and between redox-active species and counterions is ignored, the net movement of charge will be driven solely by the concentration gradient of the charged redox-active species. In this case, a Fickian-like diffusion model can be adopted to interpret the charge transport properties, and the corresponding electron diffusion coefficient, *D*_*e*_, is then determined by the rate of the electron self-exchange reaction between adjacent redox-active species.^22^
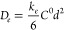
where *k*_*e*_ is the second order self-exchange rate constant, *C*^*0*^ is the total concentration of redox-active species, and *d* is the average electron hopping distance.

In real-life RC-MOFs, the situation is considerably more complex, most importantly because the unrestricted diffusion of charge balancing counterions that accompanies the electron hopping transport is physically impeded by limited pore spaces. Experimentally, apparent electron diffusion coefficient, *D*_*e*_^*app*^, are often determined in transient electrochemical experiments like step-potential chronoamperometry to quantify the charge transport properties of a RC-MOF. This large perturbation technique will create macroscopic concentration gradients of redox-active species across the MOF film. On short time scales, the semi-infinite diffusion condition is fulfilled, i.e. the electron diffusion layer thickness is small compared to the MOF film thickness (see above).^11,13,23^ In this case, the measured transient current response will be proportional to , and the apparent electron diffusion coefficient, *D*_*e*_^*app*^, can be obtained from the Cottrell equation:
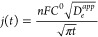
where *F* is Faraday’s constant, *n* is the number of electrons transferred between pairs of redox-active species, and *j*(*t*) is the time-dependent current density.

Alternatively to following the current response, changes in redox states of the involved redox-active linkers that are induced during the step-potential experiments may result in UV/vis absorbance changes that can be monitored by spectroelectrochemistry.^1,13^ The change in absorbance at a diagnostic wavelength, Δ*A*, as a function of time can thus be used to extract *D*_*e*_^*app*^ by applying the modified Cottrell equation:^13^
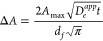
where *A*_*max*_ is the absorbance maximum, *t* is time in seconds, and *d*_*f*_ is the thickness of the MOF film.

**Figure 4:**
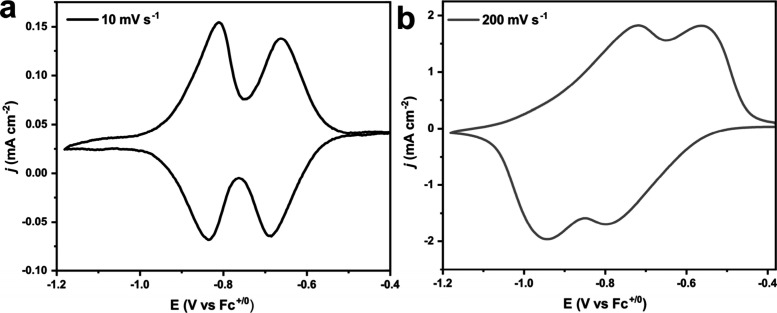
Representative CV responses of the same RC-MOF film on FTO surface at different scan rates; “surface” wave at 10 mV s^–1^ (a) and “diffusion” wave at 200 mV s^–1^ (b). Reproduced with permission from ref (14). Copyright 2023 American Chemical Society.

Lastly, CV measurements at different scan rates can be used to determine *D*_*e*_^*app*^. Specifically, at slow scan rates, the CV response of a RC-MOF film will explore the physical boundary for electron diffusion that is given by the finite thickness of the film. In other words, the time scale of the CV experiment is slower than the electron diffusion through the film (finite diffusion regime), and the voltammetric response is that of “surface” wave (Figure 4a). In this case, the reduction and reoxidation peak currents of the redox-active species will show a linear dependence on the scan rate.^2^ At higher scan rates, the situation changes and the CV response of the same RC-MOF film will take the form of a classical “diffusion” wave with large peak-to-peak separations (Figure 4b). In this case, the peak currents will be linear to the square root of the scan rate, characteristic of a diffusion-controlled process.^22^ The transition in the scan rate dependency from the finite to the semi-infinite diffusion regime can be used to calculate *D*_*e*_^*app*^ according to
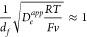
where *v* is the threshold scan rate, *R* is the universal ideal gas constant, and *T* is the temperature. It should be noted that this treatment may not be applicable to all RC-MOFs. For example, if *D*_*e*_^*app*^ is too slow, the required scan rates may be outside of what is experimentally feasible.

**Figure 5:**
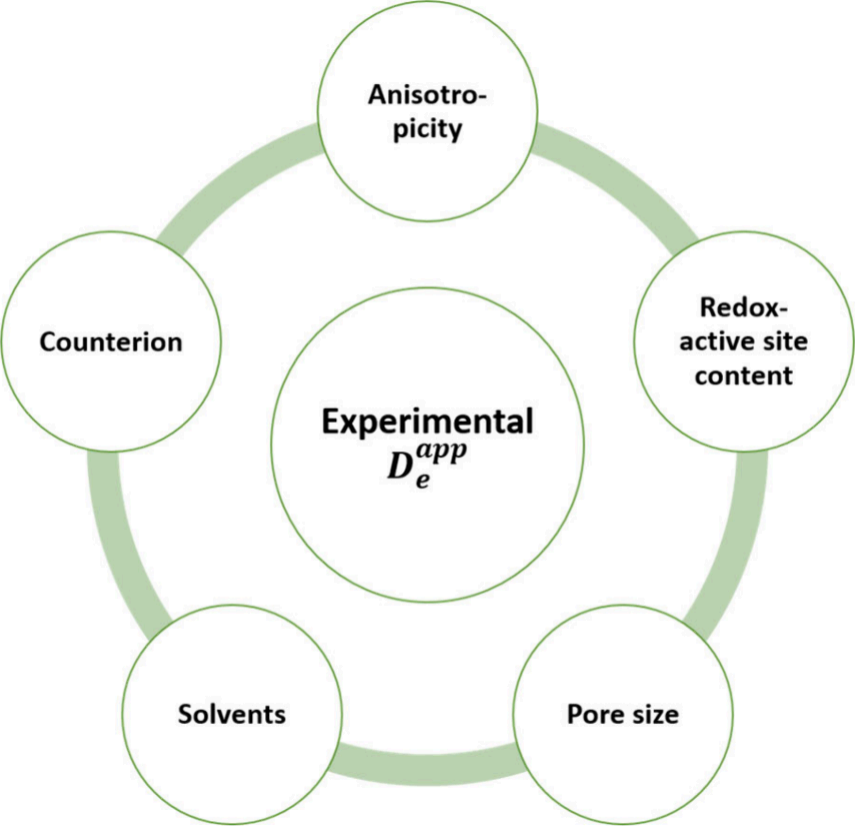
Schematic illustration of intrinsic MOF-born and extrinsic parameters that are important for experimental *D*_*e*_^*app*^.

The factors that are important for experimental *D*_*e*_^*app*^ are summarized in Figure 5. Hupp and co-workers recognized anisotropic electron hopping rates in NU-1000,^12^ a RC-MOF which operates based on the redox chemistry of its tetraphenylpyrene linkers. Having fabricated two electrodes with different orientations of NU-1000 crystals on conductive substrates, it was demonstrated that *D*_*e*_^*app*^ in the *c* direction which exhibits shorter pyrene-to-pyrene hopping distances is nearly 3500 times larger than the one in the *a,b* direction.

Related to the hopping distance-dependent charge transport, we recently reported redox-active site content-dependent charge transport in a mixed-linker MOF, Zn(NDI)_*x*_(PMDI)_*y*_ (PMDI = pyromellitic diimide bis-pyrazolate).^5^ In this MOF, two linkers of equivalent molecular size but different redox potentials are statistically distributed and it is possible to tune the hopping distance between linkers of the same identity by changing the linker ratio. It was observed that *D*_*e*_^*app*^ for electron diffusion that is promoted by the NDI/NDI^•–^ redox couple drops by 1 order of magnitude when the abundance of NDI linker is decreased from 100 to 20%. Similar phenomena have been observed for other multivariate RC-MOFs, and the idea of a percolation threshold has been introduced as a maximum distance between redox active linkers (or minimal concentration in multivariate MOFs) beyond which electron diffusion is no longer discernible.^24−26^ Additionally, Morris and co-workers have demonstrated the important role of MOF pore size on charge transport rates.^27^

Not only MOF-intrinsic, but also external factors such as the nature of the charge balancing counterions and solvent polarity impact charge transport properties and thus the measured *D*_*e*_^*app*^.^1,3,28^ For example, we have observed a decrease of *D*_*e*_^*app*^ by ∼1 order of magnitude in the Zr(NDI) thin film electrodes when TBAPF_6_ is used as supporting electrolyte instead of KPF_6_.^1^ This phenomenon is attributed to the larger size of TBA^+^ which impedes the diffusion transport within the channels of the MOF. Furthermore, when using Li^+^ as the counterion, the experimental *D*_*e*_^*app*^ drops by approximately 2 orders of magnitude when going from DMF to the less polar THF.^3^ Detailed mechanistic studies further revealed an adverse ion pairing effect on the charge transport process.

Macroscopic models demand further refinement to account for strong ion pairing interactions between the mobile counterions and fixed redox-active sites. As a result, it was predicted that the experimentally determined *D*_*e*_^*app*^ will decrease as the ion pairing association–dissociation equilibrium constant increases.^29^ We have recently introduced different microscopic models for cation-coupled electron hopping transport. Essentially, the question is whether the electron transfer from one to the next linker proceeds from a state that is ion-paired or not. As ion pairing becomes stronger, for example, in less polar solvents, charge transport may evolve to a concerted ion-electron hopping mechanism as it avoids the formation of intermediate states of high energy.

Finally, while the impact of (slow) counterion diffusion on the experimentally determined *D*_*e*_^*app*^ is well established, the importance of migration is, to the best of our knowledge, basically neglected. Typical electrolyte concentrations are in the order of 100s of mM, which is lower than linker concentrations in the RC-MOF (∼1M). At the same time, ion diffusion through the microporous MOF channels is relatively slow, giving rise to situations where electric fields may evolve during a transient chronoamperometry experiment. The direction of this electric field is such that it promotes electrons away from, and countercations toward the substrate surface. This migration is thus in the same direction as the diffusional processes, and the experimental *D*_*e*_^*app*^ will be a composite metric that contains the diffusional components, as well as supplementing migration components. To the best of our knowledge, the contribution of migration to experimentally measured *D*_*e*_^*app*^ lacks experimental evidence in RC-MOFs, but it is relatively well established in related fields. For redox-conductive polymers, the restricted mobility of counterions has been shown to lead to an electrostatic potential drop across the film.^30^ This internal electric field, in turn, accelerates electron-hopping transport beyond what would be possible from a purely diffusional scenario. Experimentally, this migration effect has been demonstrated by Murray et al. on a redox copolymer containing Os^II^/Os^III^ redox couple and Ru^II^ as diluent and ClO_4_^–^ as the counterions.^30,31^ Fitting of the data by including migration effects from an Os/Ru copolymer film resulted in *D*_*e*_^*app*^/*D*_*e*_ = 5.5, consistent with the model that the measured *D*_*e*_^*app*^ overestimates the purely diffusional transport. The effect of migration grows as the diffusivity of the counterion decreases, resulting in larger current responses and leading to a large overestimation of *D*_*e*_^*app*^. This observation is actually against the conventional conception that the overall counterion-coupled electron hopping is limited by the slower step of two contributions: electron hopping or counterion displacement. Given the fundamental similarities between redox polymers and RC-MOFs, it can be expected that migration enforced by emerging electric fields under applied potential will be a factor also in RC-MOFs. This is important, because efforts to accelerate cation transport in RC-MOFs may lead to decreased electric fields, which in turn will promote less migration. As *D*_*e*_^*app*^’s that are deduced from transient experiments have both components, the two effects may partly cancel each other out. It will be exciting to see future experimental work on RC-MOFs that address this topic.

### Steady State Charge Transport

**Figure 6:**
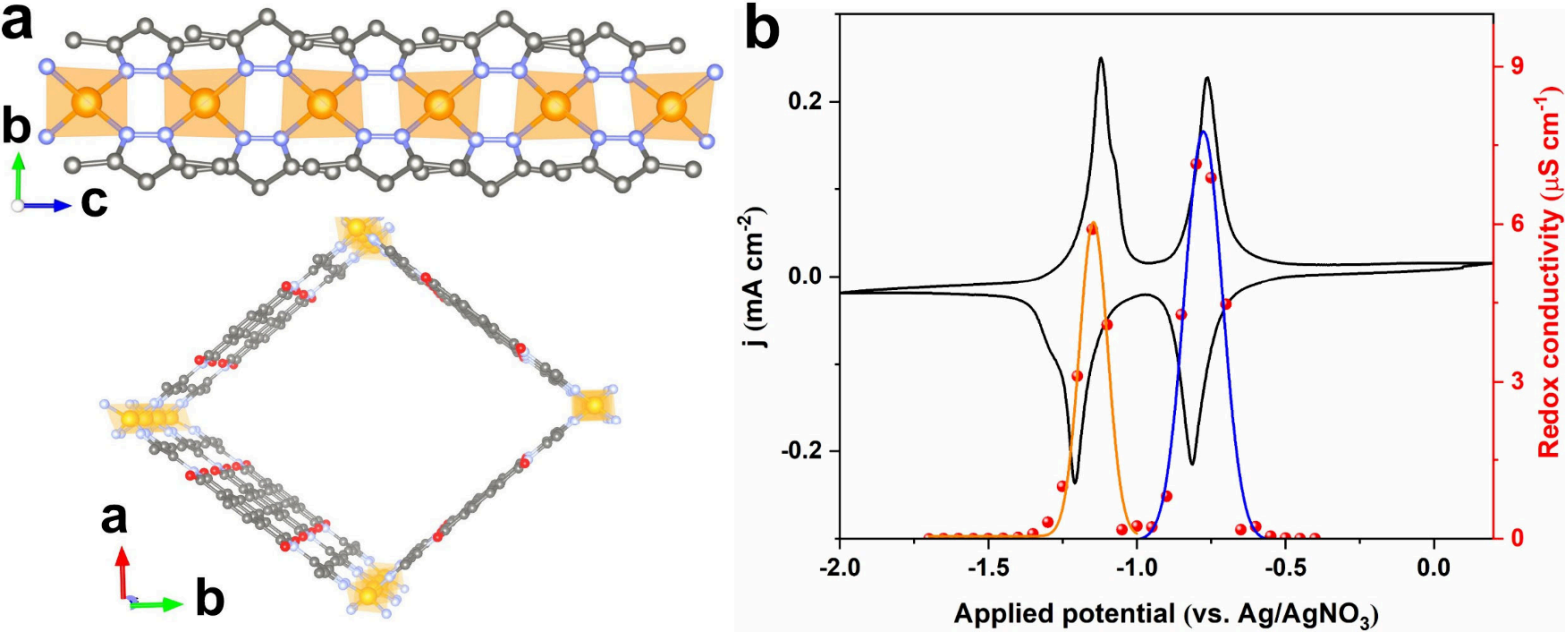
(a) Crystal structure of Zn(NDI) MOF with the projection of the pyrazolate-bridged Zn^2+^ chain (top) and viewing along the c direction (bottom). (b) Cyclic voltammetry (CV) scan (5 mV s^–1^) and steady-state redox conductivity measurement of MOF thin-films in Ar-saturated DMF with KPF_6_ as the supporting electrolyte (0.1 M). Reproduced from ref (4). Available under a CC-BY 4.0 license. Copyright 2023 Springer Nature.

Another way to access charge transport properties in RC-MOFs is through measurements of redox conductivities, σ. Despite early demonstrations by Murray and co-workers in nonporous conductive polymer films,^16^ the characteristic conductivity profile has experimentally been realized in RC-MOFs only very recently. This may relate to the fact that redox conductivity measurements demand steady state conditions and high-quality MOF films. Employing a previously published Zn(NDI) (NDI = 1,4-bis[(3,5-dimethyl)-pyrazol-4-yl]naphthalenediimide) MOF (Figure 6a) films on FTO,^32,33^ our group resolved two reversible one-electron redox processes originating from the NDI/NDI^•–^ and NDI^•–^/NDI^2–^ redox couples (Figure 6b).^4^ Using electrochemical impedance spectroscopy, two characteristic bell-shaped curves of the redox conductivity (Figure 6b) could be demonstrated for the first time. Exactly aligned to the prediction, maximum redox conductivity was observed at the formal potentials of the NDI-based redox pairs. The conductivity differences between single oxidation state films and 50:50 mixed redox state films can be up to 10000-fold, spanning the regimes of insulators and semiconductors.

In line with the transient state *D*_*e*_^*app*^, the steady state redox conductivity also exhibits dependencies on factors like hopping distance, counterions, etc.^4,5^ For example, the maximum conductivities corresponding to NDI/NDI^•–^ and PMDI^•–^/PMDI^2–^ redox pairs show clear dependences on the homolinker hopping distances in the mixed-linker Zn(NDI)_*x*_(PMDI)_*y*_ MOF.^5^ Meanwhile, when switching from K^+^ as counterions to Li^+^ and TBA^+^ with stronger ion pairing effects and larger size, respectively, decreased redox conductivity was resolved for the NDI/NDI^•–^ pair in the Zn(NDI) MOF.^4^ This collective observation of hopping distance and counterion dependencies in not only steady state redox conductivities but also *D*_*e*_^*app*^’s points to the fact that identical elementary steps are involved in both scenarios, that is, the ion-coupled electron hopping transport. Nevertheless, one should keep *D*_*e*_^*app*^ separated from the redox conductivity values as the latter can differ by up to 4 orders of magnitude depending on the film redox state (Figure 8c), while *D*_*e*_^*app*^ is generally assumed to be a constant value, characterizing the diffusional nature of the redox hoping process. Also, experimental measurement of *D*_*e*_^*app*^ involves macroscopic changes of the MOF redox state and associated counterion ingress. In contrast, the overall redox composition of the MOF is almost constant under steady state redox conductivity measurements, without net ingress of counterions.

## Applications of Redox-Conductive MOFs

The unique advantage of RC-MOFs is the ability to modulate their redox states easily through external stimulus like applied potential,^1,2,4,34,35^ chemical reduction or oxidation,^36,37^ pressure,^38^ and illumination.^39^ This, in turn, brings opportunities to modulate some of the most exciting properties, of a material, including absorption and emission, conductivity, magnetism, host–guest properties, etc. Essentially, this links to a wide spectrum of potential applications, ranging from molecular sensing, small molecule storage and separation, electrochromic materials, all the way to electrocatalysis and energy storage, switchable conductor, field-effect transistors and reconfigurable electronics, etc.^7,9,10,25,40−42^ Here, we highlight a few examples from our group.

**Figure 7:**
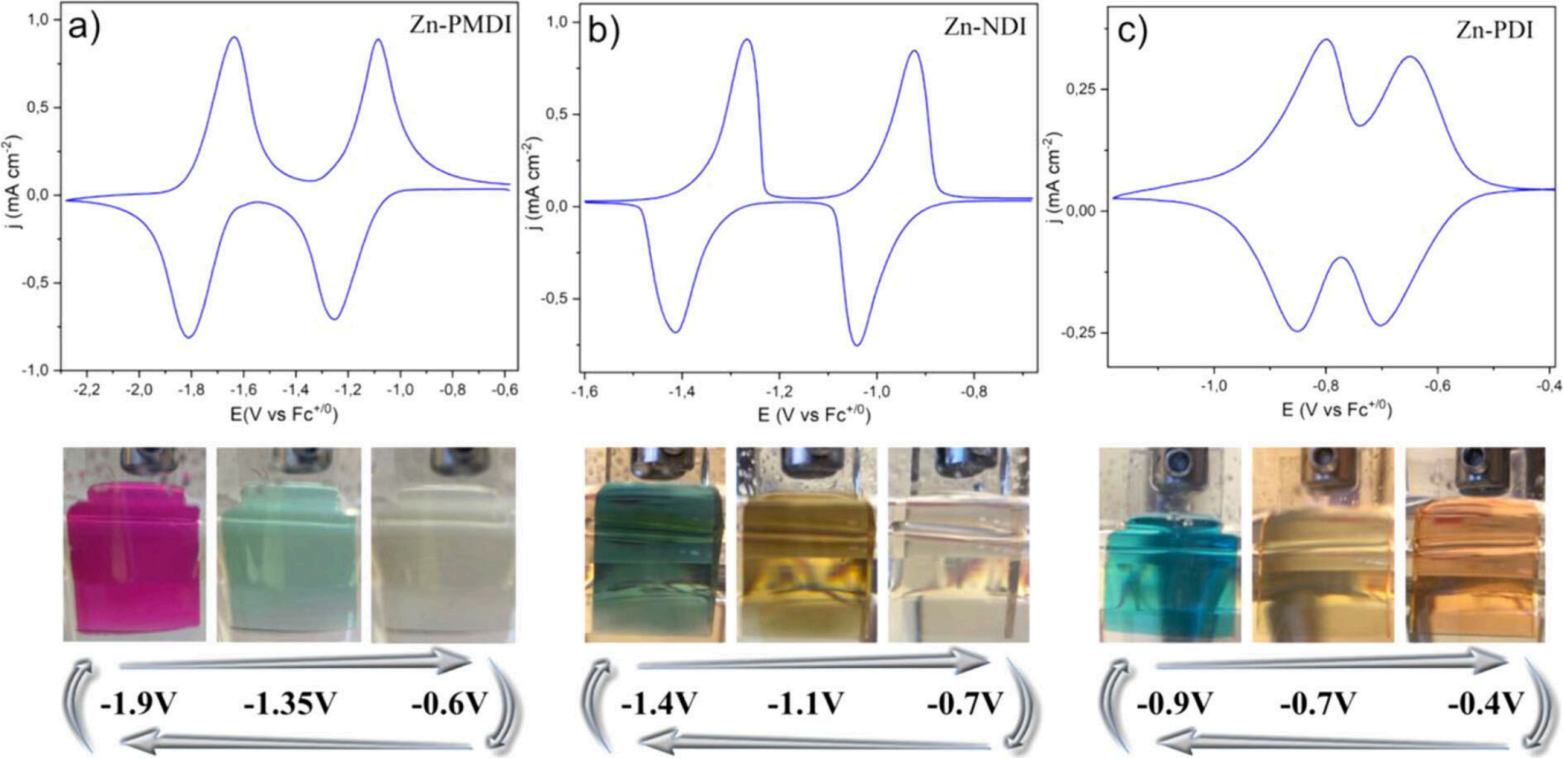
CVs and corresponding electrochromic properties (bottom panel) of Zn(PMDI) (a), Zn(NDI) (b), and Zn(PDI) (c) MOF films. Reproduced with permission from ref (14). Copyright 2023 American Chemical Society.

If at least one of the redox states of a RC-MOF has a distinct color, applications as electrochromic materials become appealing research targets.^43^ This was recognized quite early, and pioneering works were reported more than 10 years ago.^33^ Ourselves, we have reported an isoreticular series of Zn^2+^-pyrazolate MOFs, Zn(XDI) (X = PM (pyromellitic), N (naphthalene), or P (pyrylene); DI = diimide), where distinct electronic and electrochemical properties were obtained by virtue of enlarged π-systems of the constituting linkers. When grown on FTO substrates, three different redox-states of each of the Zn(XDI) MOFs, all of which exhibit distinct colors, can reversibly be accessed (Figure 7).^14^ Thanks to the discrete molecular nature of the XDI linkers in this RC-MOF, capacitive processes are kept to a minimum and Faradaic transformations are promoted, giving rise to record high coloration efficiencies of 941 cm^2^ C^–1^, a high optical contrast of 96.4% and fast switching times for the Zn(PDI) film (at 746 nm). The films were shown to be stable for at least 150 cycles, making these materials appealing candidates for electrochromic applications.

### (Photo)electrocatalysis with Coupled or Mediated Charge Transport

**Figure 8:**
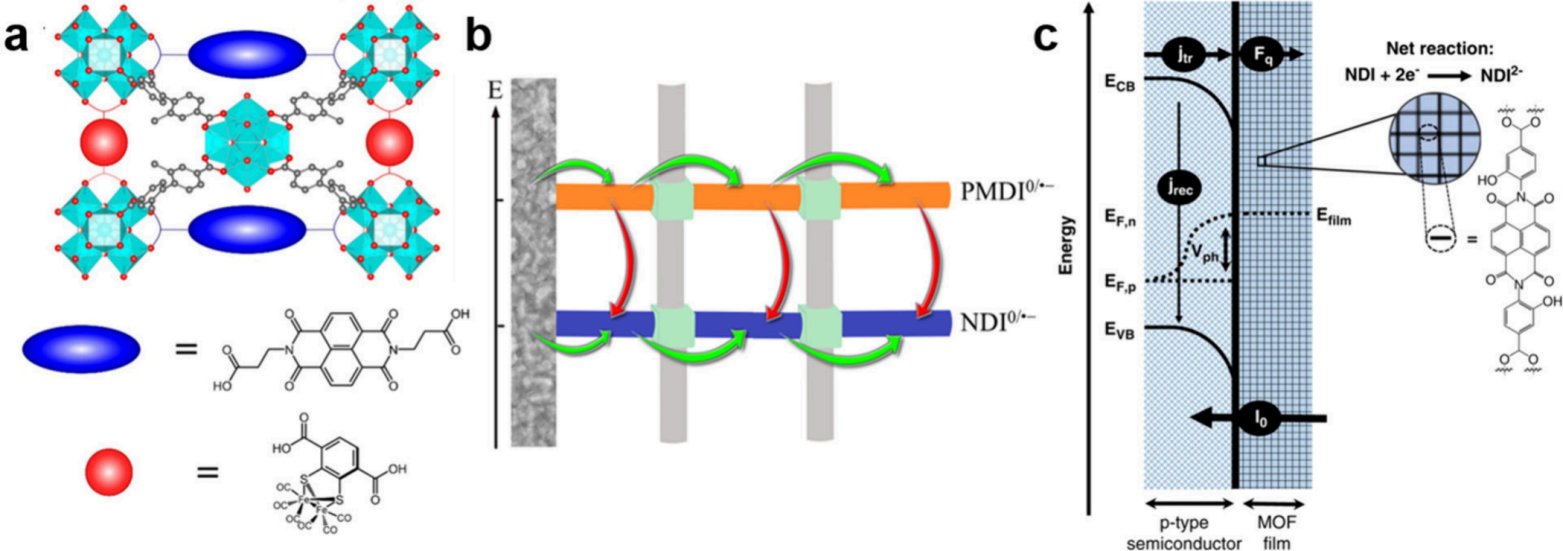
(a) Biomimetic redox-conductive PCN-700-based MOF with NDI linkers as electron mediators and [FeFe] hydrogenase as active site models for HER. (b) Schematic representation of a multivariate RC-MOF with two diffusional electron hopping transport channels (green arrows) that communicate through thermodynamically driven electron transfers from the higher to the lower energy channel. (c) Schematic representation of surface grown Zr(NDI) on p-type semiconductor surfaces. Reproduced with permission from refs (5, 48, 50). Copyright 2021, 2024 American Chemical Society and available under a CC-BY 4.0 license; 2020 Springer Nature.

When designing RC-MOFs for redox catalysis, one major task is to ensure that electron transport through the MOF is fast in comparison to the catalytic reaction, i.e. to avoid situations where catalysis occurs only in a thin reaction layer.^44^ We have published a quantitative kinetic model for diagnosing surface versus bulk reactivity for molecular catalysis in a RC-MOFs.^45^ In general, the redox-active components can act as both electron hopping units and catalytic site. This coupled strategy has been demonstrated, for example, on MOFs with porphyrin-based linkers,^13,46^ and we contributed with the redox-conductive UU-100(Co), where the metallo-cobaloxime linker both mediates electron hopping and catalyzes the hydrogen evolution reaction (HER).^2^ It is also possible to decouple the two processes, thereby allowing for the isolated optimization of both processes. Such a strategy mimics the situation in Nature’s [FeFe] hydrogenases (H_2_ases), the most active enzyme for the HER. In this enzyme, the active site is “wired” to the enzyme surface through a series of FeS clusters that effectively mediate electron transport to the active site.^47^ Despite demanding high synthetic control, the molecular nature of RC-MOFs allows us to engineer electron flow in similar ways. We have recently been able to construct a redox-conductive version of PCN-700 into which both redox-active mediators and catalytic Fe_2_ units that mimic the active sites of H_2_ases have been incorporated (Figure 8a).^48^ While the functional components were energy-matched to allow for the mediated electron transport to the Fe_2_ sites, electrocatalytic HER activity was plagued by restricted space for counterion diffusion due to physical limitations within this framework. Later, the concept was extended to a redox-conductive NU-1000-based MOF where tetraphenylpyrene linker had partially been replaced by a geometrically analogous tetratopic Ru-based metallo-linker during solvothermal synthesis.^49^ It was observed that the pyrene-based linkers can mediate oxidative equivalents to the Ru sites to drive the catalytic oxygen evolution reaction (OER), but they suffer from instability under the oxidizing aqueous conditions.

The presence of at least two linkers that are redox-active within a narrow potential window creates a complex network of electron hopping transport and electron transfer pathways. When present in sufficient abundance in the MOF, each linker species can support its own diffusional electron hopping transport, using the linkers’ unique redox chemistry. If the MOF contains two linkers with discrete redox chemistry, two channels will exist, both of which promote the diffusional electron transport by hopping between linkers of the same type (green arrows in Figure 8b). In addition, different redox potentials of the two channels result in a driving force for the electron to transfer from the higher energy “diffusional” channel to the lower one, reminiscent to the situation in the H_2_ases where the electron is transferred from the FeS clusters to the active site. We have recently been able to demonstrate all of these processes, homolinker diffusional electron transport, and thermodynamically driven heterolinker electron transfer by operando spectroelectrochemistry studies in a model mixed-linker Zn(NDI)_*x*_(PMDI)_*y*_ MOF (Figure 8b).^5^

Going beyond electrocatalysis, we have also demonstrated the possibility of growing redox-conductive Zr(NDI) MOF on some classical p-type semiconductors for photoelectrochemical applications (Figure 8c).^50^ In this case, the surface grown MOF film can quickly capture photogenerated electrons by redox reactions of the redox-active NDI linkers (Figure 8c). Therefore, the detrimental surface recombination processes can be greatly suppressed, and the operational photovoltages of the underlying semiconductors can be promoted by as much as 700 mV on GaP(100) surfaces, which is among the highest values reported for GaP in photoelectrochemical applications. Owning to the unique transport properties of RC-MOFs, photocurrents can be limited by the diffusional electron transport characteristics of the MOF under high light intensities. By changing the redox-active linkers to include redox-catalytic function, new RC-MOF based platforms for energy-related^51^ as well as organic redox transformations can be envisaged.

## Conclusions and Outlook

RC-MOFs are a unique class of porous materials where electron propagation is realized by self-exchange reactions between neighboring redox-active units that differ in their oxidation state. Due to their electronic isolation, the elementary electron hopping step is coupled to the movement of charge balancing counterions. These features are fundamentally different from band-type conductive MOFs, and as a function of redox states, characteristic bell-shaped redox-conductivity profiles are expected for RC-MOFs (Figure 2b). In this Account, we have first introduced characteristics of RC-MOFs, strategies to construct them, and experimental diagnostics for verification. Specifically, retaining molecular characters of the redox-active organic linkers or metallo-linkers is the key. We have presented techniques to characterize macroscopic charge transport properties in RC-MOFs through measuring the apparent electron diffusion coefficient, *D*_*e*_^*app*^. A wide range of MOF-related structural factors as well as experimental parameters that are important for charge transport have been identified by measuring *D*_*e*_^*app*^. Also, the theoretical limitations of the coefficient, its modifications, and complications from interactions on the molecular level were discussed. Further, we have introduced the unique charge transport properties in the steady state, i.e., the bell-shaped redox conductivity curve, as a function of applied potential. Lastly, the applications of RC-MOFs have been briefly summarized with selected highlights for electrocatalysis and electrochromic materials.

With the vast possibilities that are given to us by molecular chemistry, many more RC-MOFs with innovative metallo-linkers and organic redox-active units will be designed and prepared in the future. Meanwhile, the understanding of charge transport properties in such RC-MOFs has just begun, and the nature of ion-coupled electron hopping processes and its influencing factors at play, in particular, the role of migration, demand extensive research. Also, mechanistic studies on both macroscopic and microscopic (molecular) level are crucial to bridge the transient state (*D*_*e*_^*app*^) with steady state experiments (redox conductivity, σ). In essence, all of these efforts will contribute to the successful application of RC-MOFs in existing and emerging areas.

## References

[ref1] Johnson B. A.; Bhunia A.; Fei H.; Cohen S. M.; Ott S. Development of a UiO-Type Thin Film Electrocatalysis Platform with Redox-Active Linkers. J. Am. Chem. Soc. 2018, 140 (8), 2985–2994. 10.1021/jacs.7b13077. 29421875 PMC6067658

[ref2] Roy S.; Huang Z.; Bhunia A.; Castner A.; Gupta A. K.; Zou X.; Ott S. Electrocatalytic Hydrogen Evolution from a Cobaloxime-Based Metal-Organic Framework Thin Film. J. Am. Chem. Soc. 2019, 141 (40), 15942–15950. 10.1021/jacs.9b07084. 31508946 PMC6803166

[ref3] Castner A. T.; Su H.; Svensson Grape E.; Inge A. K.; Johnson B. A.; Ahlquist M. S. G.; Ott S. Microscopic Insights into Cation-Coupled Electron Hopping Transport in a Metal-Organic Framework. J. Am. Chem. Soc. 2022, 144 (13), 5910–5920. 10.1021/jacs.1c13377. 35325542 PMC8990995

[ref4] Li J.; Kumar A.; Johnson B. A.; Ott S. Experimental manifestation of redox-conductivity in metal-organic frameworks and its implication for semiconductor/insulator switching. Nat. Commun. 2023, 14 (1), 4388 10.1038/s41467-023-40110-6. 37474545 PMC10359279

[ref5] Li J.; Kumar A.; Ott S. Diffusional Electron Transport Coupled to Thermodynamically Driven Electron Transfers in Redox-Conductive Multivariate Metal-Organic Frameworks. J. Am. Chem. Soc. 2024, 146 (17), 12000–12010. 10.1021/jacs.4c01401. 38639553 PMC11066865

[ref6] Hendon C. H.; Rieth A. J.; Korzynski M. D.; Dinca M. Grand Challenges and Future Opportunities for Metal-Organic Frameworks. ACS Cent. Sci. 2017, 3 (6), 554–563. 10.1021/acscentsci.7b00197. 28691066 PMC5492414

[ref7] Stassen I.; Burtch N.; Talin A.; Falcaro P.; Allendorf M.; Ameloot R. An updated roadmap for the integration of metal-organic frameworks with electronic devices and chemical sensors. Chem. Soc. Rev. 2017, 46 (11), 3185–3241. 10.1039/C7CS00122C. 28452388

[ref8] Johnson B. A.; Beiler A. M.; McCarthy B. D.; Ott S. Transport Phenomena: Challenges and Opportunities for Molecular Catalysis in Metal-Organic Frameworks. J. Am. Chem. Soc. 2020, 142 (28), 11941–11956. 10.1021/jacs.0c02899. 32516534 PMC7366383

[ref9] D’Alessandro D. M. Exploiting redox activity in metal-organic frameworks: concepts, trends and perspectives. Chem. Commun. (Camb.) 2016, 52 (58), 8957–8971. 10.1039/C6CC00805D. 26988560

[ref10] Xie L. S.; Skorupskii G.; Dinca M. Electrically Conductive Metal-Organic Frameworks. Chem. Rev. 2020, 120 (16), 8536–8580. 10.1021/acs.chemrev.9b00766. 32275412 PMC7453401

[ref11] McCarthy B. D.; Beiler A. M.; Johnson B. A.; Liseev T.; Castner A. T.; Ott S. Analysis of Electrocatalytic Metal-Organic Frameworks. Coord. Chem. Rev. 2020, 406, 213137 10.1016/j.ccr.2019.213137. 32499663 PMC7272229

[ref12] Goswami S.; Hod I.; Duan J. D.; Kung C. W.; Rimoldi M.; Malliakas C. D.; Palmer R. H.; Farha O. K.; Hupp J. T. Anisotropic Redox Conductivity within a Metal-Organic Framework Material. J. Am. Chem. Soc. 2019, 141 (44), 17696–17702. 10.1021/jacs.9b07658. 31608628

[ref13] Ahrenholtz S. R.; Epley C. C.; Morris A. J. Solvothermal preparation of an electrocatalytic metalloporphyrin MOF thin film and its redox hopping charge-transfer mechanism. J. Am. Chem. Soc. 2014, 136 (6), 2464–2472. 10.1021/ja410684q. 24437480

[ref14] Kumar A.; Li J.; Inge A. K.; Ott S. Electrochromism in Isoreticular Metal-Organic Framework Thin Films with Record High Coloration Efficiency. ACS Nano 2023, 17 (21), 21595–21603. 10.1021/acsnano.3c06621. 37851935 PMC10655172

[ref15] Costentin C.; Nocera D. G. Dual-Phase Molecular-like Charge Transport in Nanoporous Transition Metal Oxides. J. Phys. Chem. C 2019, 123 (3), 1966–1973. 10.1021/acs.jpcc.8b10948.

[ref16] Chidsey C. E. D.; Murray R. W. Redox Capacity and Direct Current Electron Conductivity in Electroactive Materials. J. Phys. Chem. 1986, 90, 1479–1484. 10.1021/j100398a051.

[ref17] Kung C. W.; Goswami S.; Hod I.; Wang T. C.; Duan J.; Farha O. K.; Hupp J. T. Charge Transport in Zirconium-Based Metal-Organic Frameworks. Acc. Chem. Res. 2020, 53 (6), 1187–1195. 10.1021/acs.accounts.0c00106. 32401008

[ref18] Park J. G.; Aubrey M. L.; Oktawiec J.; Chakarawet K.; Darago L. E.; Grandjean F.; Long G. J.; Long J. R. Charge Delocalization and Bulk Electronic Conductivity in the Mixed-Valence Metal-Organic Framework Fe(1,2,3-triazolate)(2)(BF(4)) (x). J. Am. Chem. Soc. 2018, 140 (27), 8526–8534. 10.1021/jacs.8b03696. 29893567

[ref19] Aubrey M. L.; Wiers B. M.; Andrews S. C.; Sakurai T.; Reyes-Lillo S. E.; Hamed S. M.; Yu C. J.; Darago L. E.; Mason J. A.; Baeg J. O.; Grandjean F.; Long G. J.; Seki S.; Neaton J. B.; Yang P.; Long J. R. Electron delocalization and charge mobility as a function of reduction in a metal-organic framework. Nat. Mater. 2018, 17 (7), 625–632. 10.1038/s41563-018-0098-1. 29867169

[ref20] Cavka J. H.; Jakobsen S.; Olsbye U.; Guillou N.; Lamberti C.; Bordiga S.; Lillerud K. P. A New Zirconium Inorganic Building Brick Forming Metal Organic Frameworks with Exceptional Stability. J. Am. Chem. Soc. 2008, 130 (42), 13850–13851. 10.1021/ja8057953. 18817383

[ref21] Kung C.-W.; Wang T. C.; Mondloch J. E.; Fairen-Jimenez D.; Gardner D. M.; Bury W.; Klingsporn J. M.; Barnes J. C.; Van Duyne R.; Stoddart J. F.; Wasielewski M. R.; Farha O. K.; Hupp J. T. Metal–Organic Framework Thin Films Composed of Free-Standing Acicular Nanorods Exhibiting Reversible Electrochromism. Chem. Mater. 2013, 25 (24), 5012–5017. 10.1021/cm403726v.

[ref22] Andrieux C.P.; Saveant J.M. Electron Transfer Through Redox Polymer Films. J. Electroanal. Chem. 1980, 111, 377–381. 10.1016/S0022-0728(80)80058-1.

[ref23] Bard A. J.; Faulkner L. R. Electrochemical Methods: Fundamental and Applications; 2nd ed.; John Wiley & Sons, Inc., 2001; pp 613–622.

[ref24] Lin S.; Pineda-Galvan Y.; Maza W. A.; Epley C. C.; Zhu J.; Kessinger M. C.; Pushkar Y.; Morris A. J. Electrochemical Water Oxidation by a Catalyst-Modified Metal–Organic Framework Thin Film. ChemSusChem 2017, 10 (3), 514–522. 10.1002/cssc.201601181. 27976525

[ref25] Lin S.; Usov P. M.; Morris A. J. The role of redox hopping in metal-organic framework electrocatalysis. Chem. Commun. (Camb.) 2018, 54 (51), 6965–6974. 10.1039/C8CC01664J. 29809219

[ref26] Mohammad-Pour G. S.; Hatfield K. O.; Fairchild D. C.; Hernandez-Burgos K.; Rodriguez-Lopez J.; Uribe-Romo F. J. A Solid-Solution Approach for Redox Active Metal-Organic Frameworks with Tunable Redox Conductivity. J. Am. Chem. Soc. 2019, 141 (51), 19978–19982. 10.1021/jacs.9b10639. 31789028

[ref27] Cai M.; Loague Q.; Morris A. J. Design Rules for Efficient Charge Transfer in Metal-Organic Framework Films: The Pore Size Effect. J. Phys. Chem. Lett. 2020, 11 (3), 702–709. 10.1021/acs.jpclett.9b03285. 31917577

[ref28] Celis-Salazar P. J.; Cai M.; Cucinell C. A.; Ahrenholtz S. R.; Epley C. C.; Usov P. M.; Morris A. J. Independent Quantification of Electron and Ion Diffusion in Metallocene-Doped Metal-Organic Frameworks Thin Films. J. Am. Chem. Soc. 2019, 141 (30), 11947–11953. 10.1021/jacs.9b03609. 31271285

[ref29] Saveant J. M. Electron hopping between localized sites: effect of ion pairing on diffusion and migration; general rate laws and steady-state responses. J. Phys. Chem. 1988, 92 (15), 4526–4532. 10.1021/j100326a054.

[ref30] Andrieux C. P.; Saveant J. M. Electroneutrality coupling of electron hopping between localized sites with electroinactive counterion displacement. 1. Potential-step plateau currents. J. Phys. Chem. 1988, 92 (23), 6761–6767. 10.1021/j100334a053.

[ref31] Facci J. S.; Schmehl R. H.; Murray R. W. Effect of Redox Site Concentration on the Rate of Electron Transport in a Redox Copolymer Film. J. Am. Chem. Soc. 1982, 104, 4959–4960. 10.1021/ja00382a043.

[ref32] Wade C. R.; Corrales-Sanchez T.; Narayan T. C.; Dincă M. Postsynthetic tuning of hydrophilicity in pyrazolate MOFs to modulate water adsorption properties. Energy Environ. Sci. 2013, 6 (7), 2172–2177. 10.1039/c3ee40876k.

[ref33] Wade C. R.; Li M.; Dinca M. Facile deposition of multicolored electrochromic metal-organic framework thin films. Angew. Chem., Int. Ed. Engl. 2013, 52 (50), 13377–13381. 10.1002/anie.201306162. 24133021

[ref34] Qu L.; Iguchi H.; Takaishi S.; Habib F.; Leong C. F.; D’Alessandro D. M.; Yoshida T.; Abe H.; Nishibori E.; Yamashita M. Porous Molecular Conductor: Electrochemical Fabrication of Through-Space Conduction Pathways among Linear Coordination Polymers. J. Am. Chem. Soc. 2019, 141 (17), 6802–6806. 10.1021/jacs.9b01717. 30998332

[ref35] Monnier V.; Odobel F.; Diring S. Exploring the Impact of Successive Redox Events in Thin Films of Metal-Organic Frameworks: An Absorptiometric Approach. J. Am. Chem. Soc. 2023, 145 (35), 19232–19242. 10.1021/jacs.3c04114. 37615947

[ref36] Liu L.; DeGayner J. A.; Sun L.; Zee D. Z.; Harris T. D. Reversible redox switching of magnetic order and electrical conductivity in a 2D manganese benzoquinoid framework. Chem. Sci. 2019, 10 (17), 4652–4661. 10.1039/C9SC00606K. 31123575 PMC6495699

[ref37] Evans A. M.; Collins K. A.; Xun S.; Allen T. G.; Jhulki S.; Castano I.; Smith H. L.; Strauss M. J.; Oanta A. K.; Liu L.; Sun L.; Reid O. G.; Sini G.; Puggioni D.; Rondinelli J. M.; Rajh T.; Gianneschi N. C.; Kahn A.; Freedman D. E.; Li H.; Barlow S.; Rumbles G.; Bredas J. L.; Marder S. R.; Dichtel W. R. Controlled n-Doping of Naphthalene-Diimide-Based 2D Polymers. Adv. Mater. 2022, 34, e2101932 10.1002/adma.202101932. 34850459

[ref38] Chong S.; Rogge S. M. J.; Kim J. Tunable Electrical Conductivity of Flexible Metal–Organic Frameworks. Chem. Mater. 2022, 34 (1), 254–265. 10.1021/acs.chemmater.1c03236.

[ref39] Liu X.; Kozlowska M.; Okkali T.; Wagner D.; Higashino T.; Brenner-Weiss G.; Marschner S. M.; Fu Z.; Zhang Q.; Imahori H.; Brase S.; Wenzel W.; Woll C.; Heinke L. Photoconductivity in Metal-Organic Framework (MOF) Thin Films. Angew. Chem., Int. Ed. Engl. 2019, 58 (28), 9590–9595. 10.1002/anie.201904475. 31026369

[ref40] Calbo J.; Golomb M. J.; Walsh A. Redox-active metal–organic frameworks for energy conversion and storage. J. Mater. Chem. A 2019, 7 (28), 16571–16597. 10.1039/C9TA04680A.

[ref41] Nath A.; Asha K. S.; Mandal S. Conductive Metal-Organic Frameworks: Electronic Structure and Electrochemical Applications. Chem.—Eur. J. 2021, 27 (45), 11482–11538. 10.1002/chem.202100610. 33857340

[ref42] Kharod R. A.; Andrews J. L.; Dincă M. Teaching Metal-Organic Frameworks to Conduct: Ion and Electron Transport in Metal-Organic Frameworks. Annu. Rev. Mater. Res. 2022, 52 (1), 103–128. 10.1146/annurev-matsci-080619-012811.

[ref43] Tao C.-a.; Li Y.; Wang J. The progress of electrochromic materials based on metal–organic frameworks. Coord. Chem. Rev. 2023, 475, 214891 10.1016/j.ccr.2022.214891.

[ref44] Pullen S.; Fei H.; Orthaber A.; Cohen S. M.; Ott S. Enhanced photochemical hydrogen production by a molecular diiron catalyst incorporated into a metal-organic framework. J. Am. Chem. Soc. 2013, 135 (45), 16997–7003. 10.1021/ja407176p. 24116734 PMC3829681

[ref45] Johnson B. A.; Ott S. Diagnosing surface versus bulk reactivity for molecular catalysis within metal–organic frameworks using a quantitative kinetic model. Chem. Sci. 2020, 11 (28), 7468–7478. 10.1039/D0SC02601H. 33209240 PMC7116375

[ref46] Micheroni D.; Lan G.; Lin W. Efficient Electrocatalytic Proton Reduction with Carbon Nanotube-Supported Metal–Organic Frameworks. J. Am. Chem. Soc. 2018, 140 (46), 15591–15595. 10.1021/jacs.8b09521. 30392362

[ref47] Lubitz W.; Ogata H.; Rüdiger O.; Reijerse E. Hydrogenases. Chem. Rev. 2014, 114 (8), 4081–4148. 10.1021/cr4005814. 24655035

[ref48] Castner A. T.; Johnson B. A.; Cohen S. M.; Ott S. Mimicking the Electron Transport Chain and Active Site of [FeFe] Hydrogenases in One Metal-Organic Framework: Factors That Influence Charge Transport. J. Am. Chem. Soc. 2021, 143 (21), 7991–7999. 10.1021/jacs.1c01361. 34029060 PMC8176456

[ref49] Howe A.; Liseev T.; Gil-Sepulcre M.; Gimbert-Surinach C.; Benet-Buchholz J.; Llobet A.; Ott S. Electrocatalytic water oxidation from a mixed linker MOF based on NU-1000 with an integrated ruthenium-based metallo-linker. Mater. Adv. 2022, 3 (10), 4227–4234. 10.1039/D2MA00128D. 35693428 PMC9125567

[ref50] Beiler A. M.; McCarthy B. D.; Johnson B. A.; Ott S. Enhancing photovoltages at p-type semiconductors through a redox-active metal-organic framework surface coating. Nat. Commun. 2020, 11 (1), 5819 10.1038/s41467-020-19483-5. 33199706 PMC7669860

[ref51] Gibbons B.; Cairnie D. R.; Thomas B.; Yang X.; Ilic S.; Morris A. J. Photoelectrochemical water oxidation by a MOF/semiconductor composite. Chem. Sci. 2023, 14 (18), 4672–4680. 10.1039/D2SC06361A. 37181771 PMC10171202

